# Acupuncture at Back-Shu and Front-Mu Acupoints Prevents Gastric Ulcer by Regulating the TLR4/MyD88/NF-*κ*B Signaling Pathway

**DOI:** 10.1155/2021/8214052

**Published:** 2021-02-09

**Authors:** Li Li, Hao Zang, Yang Jiang, Yue Zhang, Shuangshuang Mu, Jiazhen Cao, Ying Qu, Zhaohui Wang, Wei Qi

**Affiliations:** ^1^School of Acupuncture-Moxibustion and Tuina, Changchun University of Chinese Medicine, Changchun 130117, Jilin, China; ^2^School of Pharmacy and Medicine, Tonghua Normal University, Tonghua 134002, Jilin, China; ^3^College of Pharmacy, Changchun University of Chinese Medicine, Changchun 130117, Jilin, China; ^4^Wuhan Servicebio Technology Co., Ltd., Wuhan 430200, Hubei, China; ^5^Bao'an Authentic TCM Therapy Hospital, Shenzhen 518101, Guangdong, China

## Abstract

**Purpose:**

To assess the preventive effects of acupuncture at back-shu and front-mu acupoints on rats with restraint water-immersion stress (RWIS)-induced gastric ulcer.

**Methods:**

Thirty-six rats were randomly divided into four groups for 10 days of treatment as follows: the normal group received no treatment; the model group received RWIS-induced gastric ulcer; the omeprazole group was administered omeprazole orally every 2 days; and the electroacupuncture group received electroacupuncture at the RN12 and BL21 acupoints every 2 days. After 10 days of treatment, except for the normal group, all rats were induced with gastric ulcer by RWIS for 3 h. The ulcer index (UI), ulcer inhibition rate, and histopathological score were calculated. We determined the levels of tumor necrosis factor (TNF)-*α* and interleukin (IL)-6 in serum, and the activities of myeloperoxidase (MPO), malondialdehyde (MDA), superoxide dismutase (SOD), nitric oxide (NO), and glutathione peroxidase (GSH-Px) in serum and gastric tissues. Protein expression of MyD88, nuclear factor (NF)-*κ*B (p65), and toll-like receptor (TLR) 4 was quantified in gastric tissues.

**Results:**

The electroacupuncture and omeprazole groups were equivalent in terms of UI, ulcer inhibition rate, and histopathological score. The serum levels of TNF-*α* and IL-6 were significantly lower in the electroacupuncture group compared with the omeprazole group (*P* < 0.05). Compared with the model group, there were significant changes in the levels of NO, MPO, GSH-Px, and MDA in all other groups, while the expression of TLR4, MyD88, and NF-*κ*B p65 in gastric tissue decreased significantly in the electroacupuncture group. The expression of TLR4 was substantially lower in the electroacupuncture group compared with the omeprazole group.

**Conclusion:**

Acupuncture at back-shu and front-mu acupoints played a role in preventing gastric ulcer by inhibiting extracellular signals, stimulating kinases in serum and gastric tissues, and activating the inhibition of the TLR4 signaling pathway.

## 1. Introduction

Gastric ulcer is one of the most common digestive diseases but is not easily cured; those that are cured commonly reoccur. The main causes of this illness are excessive psychological stress, alcohol consumption, smoking, *Helicobacter pylori* infection, nutritional deficiencies, and frequent use of nonsteroidal anti-inflammatory drugs [1]. When homeostasis is disrupted, the adaptive physiological stress response occurs as a mechanism to restore the steady state. Injury to organs and diseases including diabetes can be induced by chronic or severe stress. A classic example of organ injury caused by surgery, trauma, or sepsis is stress-induced gastric ulcer [2]. These ulcers are common in critically ill patients, in whom they cause high mortality [3–6], but they are also common in patients with milder conditions and can affect quality of life [7, 8]. How to treat stress ulcer and its complications remains a difficult clinical question [9].

Although the precise pathogenesis remains unknown, it is generally acknowledged that stress ulcer development involves the effects of inflammatory responses and oxidation imbalance, the upregulation of proinflammatory cytokines such as tumor necrosis factor (TNF)-*α* and interleukin (IL)-6, and the generation of reactive oxygen species (ROS) such as hydrogen peroxide (H_2_O_2_), hydroxyl radical, and superoxide anion. ROS generated by ischemic tissue are considered to be a mediator of stress-induced lesions [10]. ROS induce lipid peroxidation, which subsequently leads to loss of membrane fluidity, weakened membrane integrity, and eventually the loss of cellular functions.

Many chemically synthesized drugs can be used to cure and control gastric ulcers and associated issues, but none are specific for stress ulcers; moreover, they have many adverse effects that can cause more complications. Therefore, it is urgent that we find alternative therapies that are safer and more effective for stress ulcers.

In line with the theory of traditional Chinese medicine, the use of acupuncture at back-shu and front-mu acupoints is a common clinical method. RN12 and BL21 specifically correspond to the pregastric mu and posterior gastric shu acupoints, respectively. Back-shu acupoints are distributed in the waist and back and are used to treat yang (dorsal) imbalances; front-mu acupoints are distributed in the abdomen and chest, lie adjacent to internal organs, and are used to treat yin (ventral) imbalances. The distribution of these acupoints on both sides of the body corresponds to the anatomical location of the internal organs, forming relationships between groups of front-mu and back-shu acupoints [11]. It has been proven that the combination of the RN12 and BL21 acupoints is useful for treating gastric diseases in clinical practice, possibly achieved by regulating gastric movement [12], which is closely associated with the neuroendocrine-immune network [13]. However, the protective mechanism of acupuncture at RN12 and BL21 in the treatment of stress ulcers is unclear. The purposes of this research were to understand the role of acupuncture against stress ulcers in rats and to clarify the protective mechanism of acupuncture in general.

## 2. Materials and Methods

### 2.1. Animals

Adult male Wistar rats (weight range, 180–200 g) were obtained from Liaoning Changsheng Biotechnology Co., Ltd. (Liaoning, China). We adopted all animal-related policies as described in the guidelines of Changchun University of Chinese Medicine regarding the use of experimental animals. The animals were bred with free access to water and housed under a 12-hour light-dark cycle with a relative humidity of 55 ± 5% at a room temperature of 25 ± 3°C.

### 2.2. Animal Treatment

**Figure 1:**
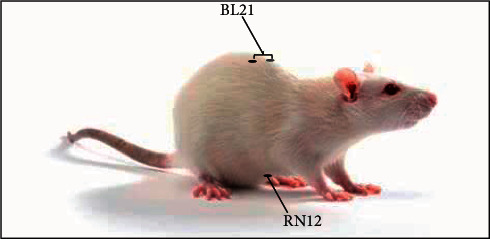
The location of the RN12 and BL21 acupoints in the rat.

The 36 rats were randomly divided into four groups of 9 rats each: normal group (untreated), model group (gastric ulcer induction with restraint plus water-immersion stress (RWIS)), omeprazole group (pretreatment with omeprazole before gastric ulcer induction), and electroacupuncture group (pretreatment with electroacupuncture before gastric ulcer induction). All experiments ran for 10 days. The groups were treated as follows: (1) normal group rats and model group rats were raised in a routine manner; (2) omeprazole group rats were given omeprazole (20 mg/kg) by gavage every 2 days; and (3) electroacupuncture group rats received electroacupuncture (2 Hz) for 10 min every 2 days. For the localization of acupuncture points in rats (Figure 1), we utilized a positioning method based on the corresponding anatomical structures in humans, as described previously [11]. The rats were anesthetized using a small animal anesthesia machine before administering electroacupuncture using acupuncture needles (Huatuo, Jiangsu, China) inserted into the acupoints corresponding to back-shu BL21 and front-mu RN12 in humans. These needles were connected to an electronic acupuncture instrument (SDZ-V apparatus, Huatuo, Jiangsu, China).

### 2.3. RWIS-Induced Gastric Ulcer Modeling

The procedure employed to induce gastric stress ulcer was previously described [14]. We aimed to minimize suffering and prevent death by drowning while carrying out this modeling. All rats except for those in the normal group were deprived of food for 48 h before modeling and were allowed to drink water freely throughout the process. The rats were placed in wire-restraint cages and immersed in a water bath up to the level of the xiphoid process for 3 h at a temperature of 22 ± 1°C.

### 2.4. Assessment of Gastric Mucosal Damage

At the end of the RWIS, the rats were anesthetized by intraperitoneal injection of sodium pentobarbital (30 mg/kg). Blood was drawn via the abdominal aorta and settled at room temperature for 20 min before separation of the serum by centrifugation at 3000 r/min at 4°C for 15 minutes; samples were preserved at −20°C until use. The stomach was removed quickly, cut along the curved side, inverted to expose the mucosal surface, and rinsed with iced sodium chloride solution. The gastric mucosal damage was evaluated macroscopically according to the Guth et al. standard [15]. The length of each lesion was measured, and the ulcer index (UI) was expressed as the amount of the length of all lesions.

For microscopic histologic examinations, the gastric tissue specimens of three rats from each group were fixed in 10% formaldehyde, dehydrated and washed, embedded by infiltration with paraffin, and sliced into 4 *μ*m thick sections. Sections were dewaxed and stained with hematoxylin and eosin. A microscopic score was assessed according to the method described previously [16]. The gastric tissues of the remaining six rats from each group were frozen in liquid nitrogen immediately after removal and stored at −80°C until use.

### 2.5. Measurement of Oxidative Stress Index Activity in Serum and Gastric Tissues

Gastric tissue was homogenized in physiological saline (ratio: 1 part tissue to 9 parts saline), then spun by centrifugation (4000 r/min for 10 min) to obtain the supernatant. Serum and gastric tissues were evaluated for myeloperoxidase (MPO), malondialdehyde (MDA), superoxide dismutase (SOD), nitric oxide (NO), and glutathione peroxidase (GSH-Px) content using assay kits according to the manufacturer's guidelines for each (Nanjing Jiancheng, China). The protein concentration in gastric tissue was determined by the bicinchoninic acid (BCA) method and was not determined for serum samples.

### 2.6. Cytokine Evaluation

The levels of the cytokines TNF-*α* and IL-6 in serum were determined using enzyme-linked immunosorbent assay kits (Thermo Fisher, America) according to the manufacturer's guidelines. The absorbance was recorded at 450 nm on a microplate spectrophotometer.

### 2.7. Western Blot Analysis

Gastric tissue was mixed with a phosphatase inhibitor cocktail, radioimmunoprecipitation assay lysis buffer, and phenylmethylsulfonyl fluoride (all from Beyotime, China), transferred to a homogenizer, homogenized in an ice bath, and then allowed to stand for 30 min; all of the samples were subjected to centrifugation (12000 g) for 10 min at 4°C. The total protein concentration of the supernatant was determined using a BCA kit, with bovine serum albumin as the standard. The supernatant (an equivalent amount of protein loaded for each sample) was loaded into a sodium dodecyl sulfate-polyacrylamide gel and separated by electrophoresis. The proteins were transferred to polyvinylidene fluoride membranes (Millipore, USA), blocked with 5% skim milk for 1 h, washed 3 times in Tris-buffered saline containing 0.1% Tween 20 (TBST), and then incubated individually with the following antibodies at 4°C overnight: primary rabbit antibodies against nuclear factor (NF)-*κ*B p65 (1 : 1000, Servicebio, China); anti-*β*-actin antibody (1 : 3000, Servicebio); anti-toll-like receptor (TLR) 4 antibody (1 : 1000, Servicebio); and anti-MyD88 antibody (1 : 1000, Bioss, China). After washing with TBST, the membranes were incubated with the secondary antibody at room temperature for 1 h. Immunoreactive bands were imaged using an enhanced chemiluminescence detection kit (Bio-Rad, USA), and Image Lab 4.1 (Bio-Rad) was applied for signal collection and densitometric image analysis.

### 2.8. Statistical Analysis

All results are expressed as mean ± standard deviation. The data were analyzed using SPSS 23.0 (IBM, Armonk, NY, USA). Statistical analysis was carried out with one-way analysis of variance accompanied by Fisher's least significant difference test. The differences in mean values between the omeprazole group and the electroacupuncture group were calculated by the *t*-test. *P* values < 0.05 were recognized as indicating statistical significance.

## 3. Results

### 3.1. Electroacupuncture Prevented Stress Ulceration

**Figure 2:**
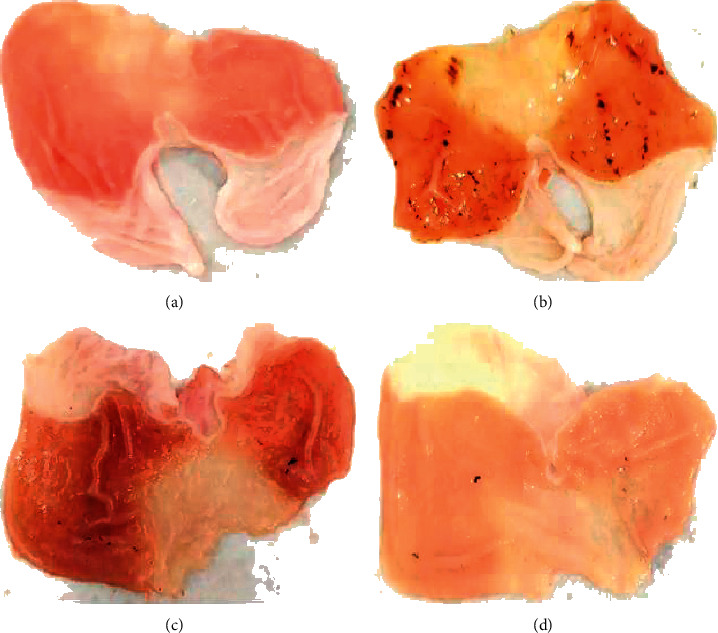
Macroscopic images of representative gastric tissues from each treatment group. (a) Normal. (b) Model. (c) Omeprazole. (d) Electroacupuncture.

As shown in Figure 2, the gastric mucosa in the normal untreated group of rats was intact and smooth, with a pale pink color. The gastric mucosa of the model group was comparatively hyperemic and less smooth, with many hemorrhagic spots of different sizes that were concentrated in the glands, with clear boundaries and no perforation. This result showed that treatment with RWIS induced dramatic gastric mucosal damage, causing obvious ulcerative lesions in the rats. In the omeprazole group, the gastric mucosa was smooth and the hemorrhagic spots were scattered. In the electroacupuncture group, the gastric mucosa was smooth and the color was lighter than any of the other groups, with the occasional observation of crater-like ulcers. This result demonstrated that pretreatment with electroacupuncture dramatically reduced the number and extent of lesions compared with the model group. Moreover, electroacupuncture showed similar effects to omeprazole.

### 3.2. Electroacupuncture Decreased the Gastric UI

The success rate of gastric ulcer modeling by RWIS was 100%. The model group had the highest UI, which was statistically significant compared with the normal group (Table 1). The UI was lower in both the omeprazole and electroacupuncture groups compared with the model group. Furthermore, there were no significant differences in UI or ulcer inhibition rate between the electroacupuncture and omeprazole groups, demonstrating that electroacupuncture pretreatment reduced the UI and that acupuncture had the same effects as omeprazole (Table 1).

### 3.3. Electroacupuncture Reduced Gastric Mucosal Tissue Lesions

**Figure 3:**
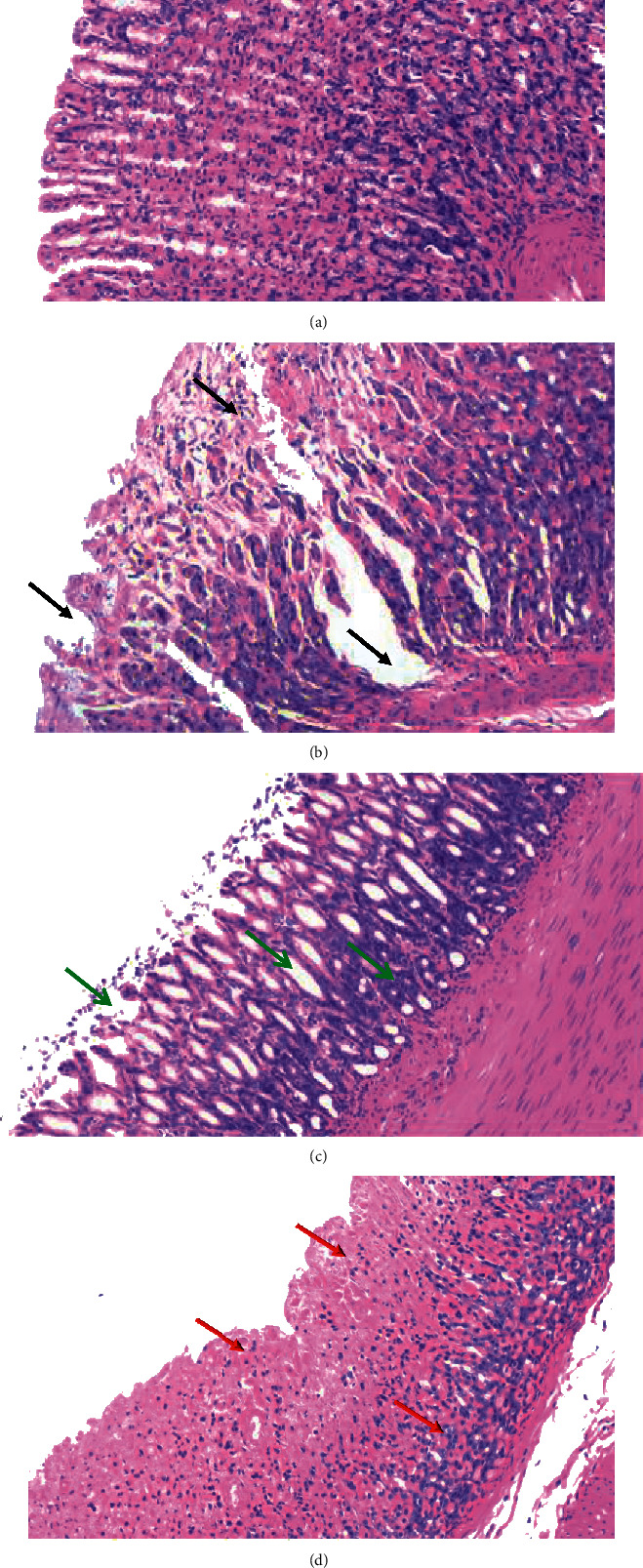
Representative histopathological photos of gastric tissue sections from each group. (a) Normal. (b) Model. (c) Omeprazole. (d) Electroacupuncture.

Histopathologic results are shown in Figure 3. In the normal group, the gastric mucosa was smooth and intact, with neat arrangement of epithelial cells, and no capillary blood congestion or inflammatory cell infiltration. In the model group, the mucosal epithelial structure was destroyed, with large numbers of dead mucosal epithelial cells (black arrows). We also observed contraction and deep staining of the cell nuclei, aggravated demyelination, disorderly arrangement of glands, and rupturing of submucosal blood vessels. Large numbers of red blood cells could be seen in the intercellular space, and there was inflammatory cell infiltration. Compared with the model group, the omeprazole group showed thinning of the mucosa and submucosa, and neat arrangement of glands, but the glandular cavity was slightly larger and there was minor infiltration of inflammatory cells (green arrows). In the electroacupuncture group, the gastric mucosal surface was comparatively less damaged, with different degrees of repair and hyperplasia (red arrows). We also observed shedding of epithelial cells in the omeprazole and electroacupuncture groups, and there was less inflammatory cell infiltration compared with the model group. Interestingly, preacupuncture treatment successfully improved the pathological features by decreasing congestion and tissue inflammation, consequently reducing the pathological scores.

**Figure 4:**
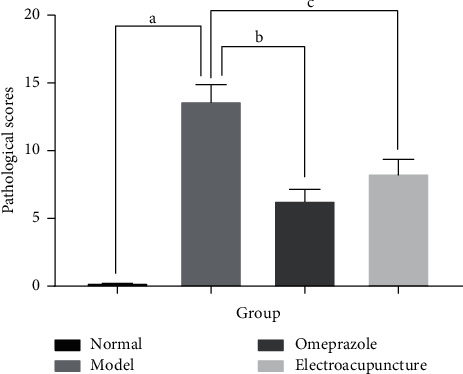
Histopathological lesion scores in each group. ^a^Model group compared with the normal group (*P* < 0.05); ^b^omeprazole group compared with the model group (*P* < 0.05); ^c^electroacupuncture group compared with the model group (*P* < 0.05).

Histopathological examination scoring (Figure 4) showed that the model group had the highest lesion score, with a significant difference compared with the normal group. Both the omeprazole group and the electroacupuncture group had significantly lower lesion scores than the model group.

### 3.4. Electroacupuncture Reduced the Level of Peroxide in Serum and Gastric Tissues

**Figure 5:**
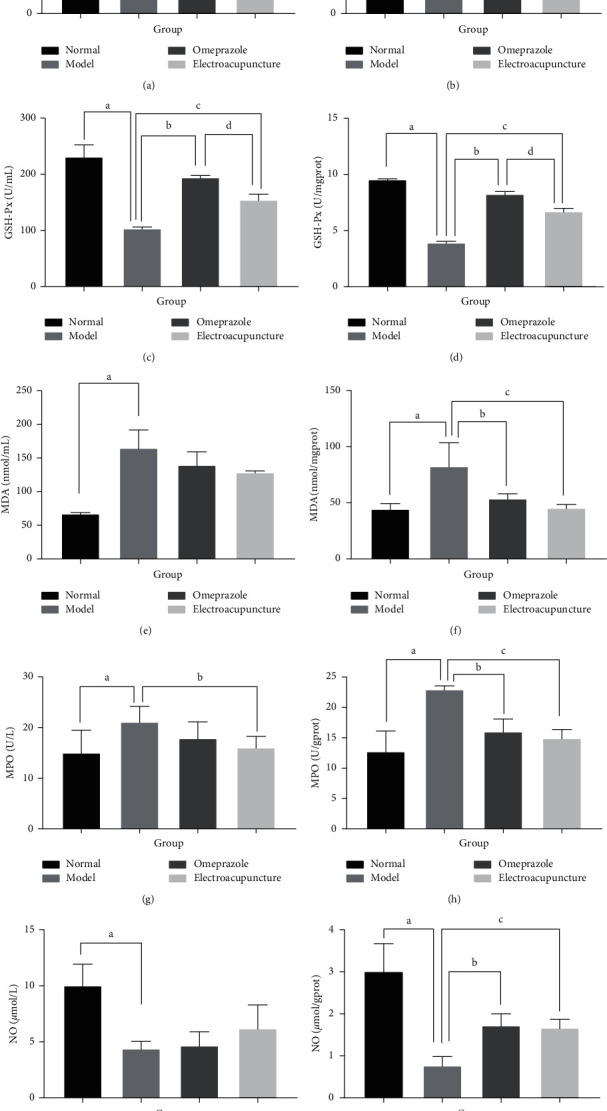
Levels of five oxidative stress indicators in serum (left panels) and gastric tissue (right panels) in each group. ^a^Model group compared with the normal group (*P* < 0.05); ^b^omeprazole group compared with the model group (*P* < 0.05); ^c^electroacupuncture group compared with the model group (*P* < 0.05); ^d^electroacupuncture group compared with the omeprazole group (*P* < 0.05).

Compared with the normal group, there was increased MDA activity in the serum and gastric tissues of the other three groups, demonstrating that the oxidative damage caused by stress ulcer induction is obvious (Figure 5). Compared with the model group, there was lower MDA activity in the serum and gastric tissues in the electroacupuncture and omeprazole groups, with the decline being more obvious in the gastric tissue. At the same time, the levels of GSH-Px and NO increased significantly. The GSH-Px content was lower in the electroacupuncture group compared with the omeprazole group, but there was no significant difference between the two in NO content.

Compared with the normal group, the MPO content of the model group increased significantly. Compared with the model group, in the electroacupuncture group, the MPO content was significantly lower in the serum and gastric tissues, while the content of MPO decreased significantly only in the gastric tissue in the omeprazole group. Conversely, the levels of SOD in the serum and gastric tissues of both the omeprazole and electroacupuncture groups showed an increasing trend. These results demonstrated that electroacupuncture and omeprazole inhibited the oxidative damage of stress ulceration to an obvious degree and that the effects were specific.

### 3.5. Electroacupuncture Reduced the Expression of Serum Inflammatory Factors

**Figure 6:**
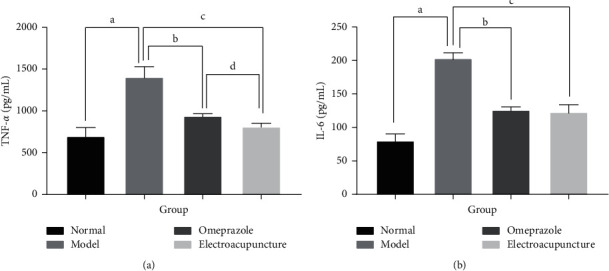
The expression of two inflammatory factors in serum in each group. ^a^Model group compared with the normal group (*P* < 0.05); ^b^omeprazole group compared with the model group (*P* < 0.05); ^c^electroacupuncture group compared with the model group (*P* < 0.05); ^d^electroacupuncture group compared with the omeprazole group (*P* < 0.05).

As shown in Figure 6, the expression of IL-6 and TNF-*α* in serum in the three induced stress ulcer groups was increased compared with the normal group, indicating that overexpression of inflammatory factors occurs during gastric mucosal injury. Compared with the model group, however, the expression of IL-6 and TNF-*α* in serum was markedly reduced in the electroacupuncture and omeprazole groups, suggesting that electroacupuncture and omeprazole can reduce the expression of related inflammatory factors in serum. The electroacupuncture group also had significantly reduced expression of TNF-*α* compared with the omeprazole group, further suggesting that pretreatment with electroacupuncture has an obvious inhibitory effect on the inflammatory response induced by stress ulcer.

### 3.6. Electroacupuncture Inhibited the Protein Expression of TLR4, MyD88, and NF-*κ*B p65

**Figure 7:**
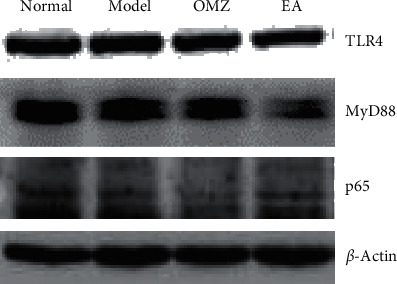
Western blotting showing representative bands of three members of the TLR4/NF-*κ*B pathway in each group. Nor: normal group; Mod: model group; OMZ: omeprazole group; EA: electroacupuncture group.

The expression levels of TLR4, MyD88, and NF-*κ*B (p65) proteins in gastric tissue in the model group were dramatically increased compared with the normal group (Figure 7 and Table 2). These proteins showed decreased expression in the omeprazole and electroacupuncture groups compared with the model group. Expression of TLR4 and MyD88 was significantly reduced in the electroacupuncture group compared with the omeprazole group, indicating that the preventive effect of electroacupuncture on stress ulcers may be exerted specifically via suppression of the TLR4/NF-*κ*B signaling pathway.

## 4. Discussion

This study showed that electroacupuncture prevented stress ulcers with effects that were comparable to those of omeprazole treatment in rats. Electroacupuncture and omeprazole may prevent stress ulcers through a combination of antioxidant and anti-inflammatory actions, and by inhibition of the TLR4/MyD88/NF-*κ*B signaling pathway.

At present, there is no satisfactory treatment for stress ulcers. Stress ulcer prophylaxis [17] is recommended, most commonly with proton pump inhibitors (PPIs) and type II histamine receptor antagonists [18]. However, the use of PPIs may increase the chance of nosocomial pneumonia, *Clostridium difficile* infections, and cardiovascular events [19].

Acupuncture is a safe treatment method with minimal impact on the environment. Electroacupuncture has been proven to relieve multiple stress-induced conditions, including irregular gastric rhythm, abnormal defecation, delayed gastric emptying, and anxiolytic-like behavioral effects [20–22]. Electroacupuncture is widely used in various gastrointestinal diseases by anti-inflammatory mechanism [23, 24].

Studies have described multiple protective mechanisms of acupuncture on gastric mucosal tissues, which include strengthening the gastric mucosal barrier, regulating gastric acid secretion and gastrointestinal hormones, improving gastric mucosal blood flow, and enhancing antagonism against free radical-induced lesions [25].

There is increasing evidence that ROS play a crucial role in the formation of stress ulcers [26]. ROS may cause direct tissue damage or induce downstream signaling pathways to mediate inflammatory damage.

Previous reports indicate that MDA is the main product of lipid peroxidation and a biomarker for oxidative stress. MPO is an important peroxidase expressed in neutrophils. MPO can be used not only as a quantitative measure of neutrophil numbers (as a reflection of neutrophil infiltration), but also as a quantitative reflection of the degree of inflammatory damage. NO is related to gastrointestinal mucosal injury and defense. NO supports the healthy function of the gastrointestinal mucosa and has a protective effect on cells. SOD is the main antioxidant against ROS and can effectively prevent gastrointestinal mucosal damage. GSH-Px further degrades H_2_O_2_ produced by SOD disproportionation [27]. The induction of stress ulcer in the model rats in this study significantly increased MDA and MPO and significantly decreased SOD, GSH-Px, and NO compared with normal, untreated rats. These results indicate that when stress ulcers occur, oxidized substances are increased, while antioxidant substances that could reduce gastric tissue damage are decreased. After pretreatment with omeprazole or electroacupuncture, SOD and NO increased and GSH-Px increased markedly in the omeprazole and electroacupuncture groups; GSH-Px was significantly higher in the omeprazole group than in the electroacupuncture group. MDA and MPO decreased, especially in gastric tissue, and there was significantly lower serum MPO in the electroacupuncture group than the model group.

Previous studies have also found that an imbalance between the production of ROS and endogenous antioxidant activity can cause oxidative stress. ROS in ulcer tissue may be derived from the infiltration of inflammatory cells [28]. Superoxide radical anions are generated by the reaction of neutrophils with lipids, eventually leading to the production of lipid peroxides [29].

Our experiments showed that the prevention of gastric ulcers induced by RWIS in rats was mainly exerted by increasing GSH-Px and decreasing MPO and MDA. Therefore, the mechanism of action of electroacupuncture in preventing gastric ulcers in rats appears to be the reduction of neutrophil infiltration and prevention of lipid peroxidation. This protective therapeutic effect was stronger in the electroacupuncture group than the omeprazole group.

Inflammation is another key mechanism that leads to the involvement of increased ROS production in gastric ulcerative injury. It is well known that gastric ulcers are the result of tissue necrosis caused by mucosal congestion. Tissue necrosis and chemokine release attract immune cells, phagocytosis of necrotic tissue, and the release of proinflammatory cytokines such as TNF-*α* and IL-6, thereby activating local endothelial cells and epithelial cells [30].

Research has shown that acupuncture can reduce TNF-*α* and IL-6 levels in serum [31, 32], as well as inhibit the degree of intracellular inflammatory response [33–35]. The results of our study showed that the levels of IL-6 and TNF-*α* were significantly, markedly higher in the model group compared with the normal group, indicating that stress ulceration increases the infiltration of proinflammatory cells. IL-6 and TNF-*α* levels were significantly lower in rats pretreated with omeprazole or electroacupuncture compared with those in the model group, and TNF-*α* was further reduced in the electroacupuncture group compared with the omeprazole group (*P* < 0.05). This observation suggests that pretreatment with electroacupuncture can inhibit inflammation and protect gastric tissue by reducing the infiltration of inflammatory cells and minimizing the secretion of proinflammatory cytokines including IL-6 and TNF-*α*.

NF-*κ*B is the main transcription factor for the regulation of inflammation. Proinflammatory factors driven by NF-*κ*B create a powerful signal that regulates immune cells involved in inflammation [36]. In the TLR4/MyD88 pathway, NF-*κ*B is a prominent downstream signaling molecule [37, 38]. MyD88 was the first member in the TLR family to be identified and is used as an adaptor by all TLRs except for TLR3; it activates NF-*κ*B to induce inflammatory cytokines [39]. The TLR4/MyD88/NF-*κ*B pathway is related to the immune response, and its activation is the key to the development of various diseases. When the signal is transferred from TLR4 to MyD88, with the continuous recruitment of IRAK4 and TRAF6, the IKK complex will be activated, causing the proteasome to destroy I*κ*B. Next, the NF-*κ*B p65 subunit is allowed to translocate into the nucleus and promote the production of proinflammatory cytokines such as TNF-*α* and IL-6 [40]. Recent research indicates that both oxidative stress and infection stress can share the same TLR signaling pathway [41–44]. Thus, it is plausible that the pathogenesis of stress-induced gastric ulcer involves the TLR4/MyD88/NF-*κ*B signal transduction pathway. Studies have reported that acupuncture exerts anti-inflammatory effects in rats with cerebral ischemia-reperfusion injury by inhibiting the TLR4/NF-*κ*B signaling pathway [45]. Our research showed that expression of TLR4, NF-*κ*B, and MyD88 was significantly, markedly increased by RWIS in the model group and significantly decreased by pretreatment with electroacupuncture, indicating that preventive electroacupuncture could inhibit the activation of TLR4/NF-*κ*B signaling induced by stress ulcer.

## 5. Conclusions

We confirmed the protective effect of pretreatment with electroacupuncture at the back-shu and front-mu acupoints on stress-induced gastric ulcers induced by RWIS in rats. Changes in the TLRs/NF-*κ*B signaling pathway revealed that acupuncture and omeprazole operate through a mechanism involving antioxidation and anti-inflammation. Acupuncture is a common clinical method for treating gastric ulcers, and our results provide a scientific basis for using acupuncture to prevent gastric ulcers.

## Figures and Tables

**Table 1:** Ulcer index and ulcer inhibition rate in each group.

Group	Ulcer index (mm^2^)	Ulcer inhibition rate (%)
Normal	—	—
Model	10.17 ± 1.77^a^	
Omeprazole	3.33 ± 0.75^b^	67.21%
Electroacupuncture	4.33 ± 0.75^c^	57.38%

^a^Model group compared with the normal group (*P* < 0.05); ^b^omeprazole group compared with the model group (*P* < 0.05); ^c^electroacupuncture group compared with the model group (*P* < 0.05).

**Table 2:** Protein expression of TLR4, MyD88, and NF-*κ*B (p65) in the gastric tissues in each group.

Group	TLR4	MyD88	NF-*κ*B(p65)
Normal	0.75 ± 0.20	0.64 ± 0.24	0.66 ± 0.06
Model	1.30 ± 0.30^a^	1.19 ± 0.22^a^	1.20 ± 0.03^a^
Omeprazole	0.88 ± 0.19^b^	0.86 ± 0.19^b^	0.77 ± 0.06^b^
Electroacupuncture	0.68 ± 0.29^c,d^	0.77 ± 0.21^c,d^	0.76 ± 0.06^c^

^a^Model group compared with the normal group (*P* < 0.05); ^b^omeprazole group compared with the model group (*P* < 0.05); ^c^electroacupuncture group compared with the model group (*P* < 0.05); ^d^electroacupuncture group compared with the omeprazole group (*P* < 0.05).

## Data Availability

The data used to support the findings of this study are included within the study and available from the corresponding author upon request.

## Abbreviations

UI: Ulcer index
IL-6: Interleukin-6
TNF-*α*: Tumor necrosis factor-*α*
SOD: Superoxide dismutase
MDA: Malondialdehyde
MPO: Myeloperoxidase
NO: Nitric oxide
GSH-Px: Glutathione peroxidase
TLR4: Toll-like receptor 4
MyD88: Myeloid differentiation primary response gene 88
NF-*κ*B: Nuclear factor kappa B
IL-1*β*: Interleukin-1*β*
ROS: Reactive oxygen species
H2O2: Hydrogen peroxide
OH: Hydroxyl radical
BCA: Bicinchoninic acid
ELISA: Enzyme-linked immunosorbent assay
PMSF: Phenylmethylsulfonyl fluoride
RIPA: Radioimmunoprecipitation assay
SDS-PAGE: Sodium dodecyl sulfate-polyacrylamide gel electrophoresis
PVDF: Polyvinylidene fluoride
TBST: Tris-buffered saline Tween
ANOVA: Analysis of variance
LSD: Least significant difference
SUP: Stress ulcer prophylaxis
PPI: Proton pump inhibitor
IRAK4: Interleukin-1 receptor-associated kinase 4
TRAF6: TNF receptor-associated factor 6
IKK: I*κ*B kinase
I*κ*B: Inhibitor of NF-*κ*B.
